# Silencing Dkk1 expression rescues dexamethasone-induced suppression of primary human osteoblast differentiation

**DOI:** 10.1186/1471-2474-11-210

**Published:** 2010-09-15

**Authors:** Joseph S Butler, Joseph M Queally, Brian M Devitt, David W Murray, Peter P Doran, John M O'Byrne

**Affiliations:** 1 Clinical Research Centre, UCD School of Medicine & Medical Science, Mater Misericordiae University Hospital, Dublin, Ireland; 2 Department of Trauma & Orthopaedic Surgery, Royal College of Surgeons in Ireland, Cappagh National Orthopaedic Hospital, Dublin, Ireland

## Abstract

**Background:**

The Wnt/β-catenin pathway is a major signaling cascade in bone biology, playing a key role in bone development and remodeling. The objectives of this study were firstly, to determine the effects of dexamethasone exposure on Wnt/β-catenin signaling at an intracellular and transcriptional level, and secondly, to assess the phenotypic effects of silencing the Wnt antagonist, Dickkopf-1 (Dkk1) in the setting of dexamethasone exposure.

**Methods:**

Primary human osteoblasts were exposed in vitro to 10^-8 ^M dexamethasone over a 72 h time course. The phenotypic marker of osteoblast differentiation was analyzed was alkaline phosphatase activity. Intracellular β-catenin trafficking was assessed using immunoflourescence staining and TCF/LEF mediated transcription was analyzed using a Wnt luciferase reporter assay. Dkk1 expression was silenced using small interfering RNA (siRNA).

**Results:**

Primary human osteoblasts exposed to dexamethasone displayed a significant reductions in alkaline phosphatase activity over a 72 h time course. Immunoflourescence analaysis of β-catenin localization demonstrated a significant reduction in intracytosolic and intranuclear β-catenin in response to dexamethasone exposure. These changes were associated with a reduction of TCF/LEF mediated transcription. Silencing Dkk1 expression in primary human osteoblasts exposed to dexamethasone resulted in an increase in alkaline phosphatase activity when compared to scrambled control.

**Conclusions:**

Wnt/β-catenin signaling plays a key role in regulating glucocorticoid-induced osteoporosis *in vitro*. Silencing Dkk1 expression rescues dexamethasone-induced suppression of primary human osteoblast differentiation. Targeting of the Wnt/β-catenin signaling pathway offers an exciting opportunity to develop novel anabolic bone agents to treat osteoporosis and disorders of bone mass.

## Background

Osteoporosis is a systemic skeletal disorder characterized by low bone mass and a progressive micro-architectural deterioration of bone tissue, leading to an increase in bone fragility and ultimately susceptibility to fracture [1]. There has been an increasing interest in elucidating the role played by developmental pathways, such as Wnt/β-catenin cascade, in bone mass regulation [2].

Wnt proteins are a family of secreted cysteine-rich glycoproteins with functions relating to cell specification, formation of the body plan, cell growth, proliferation, differentiation and apoptosis [3-5]. Wnts are defined by their sequence homology to Drosophila *w*ingless and the murine *int*-1 proto-oncogene. Human and mouse genomes have been shown to encode 19 and 18 Wnt genes, respectively [6]. Wnt proteins activate a number of distinct intracellular signal transduction pathways which have been implicated in numerous developmental and disease processes.

The role of canonical Wnt signaling in bone development and remodeling was first identified when the loss-of-function mutation of the LDL receptor-related protein (LRP5) was associated with osteoporosis psuedoglioma syndrome, a recessive disorder characterized by osteogenesis imperfecta with low bone mass and a predisposition to fractures as well as ocular defects [7,8]. Subsequently, gain-of-function mutations of the LRP5 have been shown to be associated with disorders of increased bone mass, such as Van Buchem's disease, osteopetrosis, and endosteal hyperostosis [9-11].

In this study, we focused our attention on the role played by Wnt/β-catenin signaling in glucocorticoid-induced osteoporosis. We determined the effects of dexamethasone exposure on Wnt/β-catenin signaling in primary human osteoblasts. We also examined the phenotypic effects of attenuating the expression of Dkk1, a Wnt antagonist, in primary human osteoblasts exposed to dexamethasone.

## Methods

### Cell Culture

Primary human osteoblasts from normal human hip samples were (Promocell, Heidelberg, Germany). Cells were cultured in Osteoblast Growth Medium (Promocell, Heidelberg, Germany) containing 10% FCS and antibiotics (100 IU/ml penicillin and 100 μg/ml streptomycin) at 37°C, 5% CO_2_. For all experiments primary human osteoblasts used were between 2^th ^and 5^th ^passage. Cells were treated over a 48 h time course with 10^-8 ^M Dexamethasone (Dex) (Sigma-Aldrich, Poole, UK). Institutional Review Board approval for this study was obtained from the Mater Misericordiae University Hospital Research Ethics Committee.

### Alkaline Phosphatase Activity

Alkaline phosphatase (ALP) activity was assayed by the measurement of released *p*-nitrophenol from *p*-nitrophenolphosphate (pNPP). Post-treatment cell lysates were collected by Cell Lytic MT (Sigma-Aldrich, Poole, UK) treatment and the extracted protein was frozen at -80°C for at least 2 h prior to ALP activity assay to disrupt cellular membranes. 10 μl of thawed cell lysates were incubated with 200 μl *p*NPP reagent (Sigma-Aldrich, Poole, UK) for 30 min at room temperature. ALP activity was measured at 405 nm and normalized to total protein extracted, which was measured using a bicinchoninic acid (BCA) protein assay kit (Pierce, Rochford, Illinois). One unit of ALP activity was defined as the amount required for the liberation of 1.0 μmol *p*-nitrophenol/min/mg of cellular protein.

### Immunoflourescence Staining

Beta-catenin was visualized by indirect immunocytochemistry using a mouse anti β-catenin (Santa Cruz Biotechnology, Santa Cruz, CA, USA) as the primary antibody. Primary human osteoblasts were plated on coverslips and treated with Dex-containing media. Cells were fixed in ice-cold methanol and permeabilized for ten minutes. Cells were then blocked with 10% goat serum for ten minutes at room temperature. Samples were incubated for 1 hr with primary antibody followed by a 30 min incubation with a goat anti-mouse TRITC-conjugate. Cells were viewed with a Zeiss Axioskop 40 fluorescence microscope using 20× objectives. Digital images were captured with a ProgRes C10^plus ^research digital camera (Jenoptik Laser Optik Systeme GmbH, Jena, Germany). The digital images were processed using the ProgRes CapturePro 2.1 image analysis softwear package (Jenoptik Laser Optik Systeme GmbH, Jena, Germany).

### Generation of Conditioned Media

Mouse Wnt3a overexpressing cells (L-Wnt3a) and control non-transfected L-cells were obtained from the American Type Culture Collection (ATCC, Manassas, USA) and cultured in DMEM with glutamax, 10% FBS, 100 IU/ml penicillin and 100 μg/ml streptomycin. L-Wnt3a culture medium was supplemented with 400 μg/ml geneticin to maintain selective pressure. Conditioned medium from L-Wnt3a and control L-cells was collected according to the manufacturer's instructions. Briefly, cells were passaged 1:10 in 8 ml medium without geneticin and left to grow for 10 days. Medium was collected from each cell line and replaced with 8 ml fresh medium for a further 3 days. This second batch of medium was then collected and the cells discarded. First and second batches were combined, sterile filtered (0.2 μm) and stored at -20°C until required.

### β-Catenin/TCF Transcription Reporter Assay

Primary human osteoblasts plated in 24-well plates at 2 × 10^4^/cm^2 ^were transiently transfected with the Wnt-luciferase reporter construct pBAR (1 μg total; Dr. RT. Moon, University of Washington, Seattle, WA, USA) and the control reporter pfuBAR (1 μg total; Dr. RT. Moon, University of Washington, Seattle, WA, USA) using GeneJuice (Novagen, Madison, WI, USA). The β-catenin activated reporter (pBAR) contains 12 TCF binding sites (5'-AGATCAAAGG-3') separated by distinct 5 base linkers. These elements are directly upstream of a minimal thymidine kinase promoter which then drives the expression of firefly luciferase. The control reporter pfuBAR is identical to its sister reporter with the exception of a two base substitution in each TCF binding site. Both reporters contain a separate PGK promoter that constitutively drives the expression of a puromycin resistance gene, and both are in a lentiviral platform. Co-transfection with 0.5 μg of the internal control reporter pSL9EF1a(P)RLUC (Dr. RT. Moon, University of Washington, Seattle, WA, USA), driving constitutive expression of Renilla luciferase, was systematically performed to normalize for transfection efficiency. Sixteen hours after transfection, cells were washed and cultured for 48 hr in Dex-containing media with 2% FCS, in the presence or absence Wnt3a-conditioned media. Cells were lysated, and luciferase assays were performed with the Dual Luciferase Assay Kit (Promega, Madison, WI, USA) according to manufacturer's instructions. 10 μl of cell lysates were first assayed for firefly luciferase and then *Renilla *luciferase activity. Firefly luciferase activity was normalized to *Renilla *luciferase activity.

### siRNA-mediated Dkk1 Gene Silencing

Predesigned short interfering RNA (siRNA) targeting human Dkk1 (Hs_DKK1_1) and a control scrambled RNA targeting a sequence not sharing homology with the human genome (AllStars Negative Control) were purchased (Qiagen, Crawley, UK). Primary human osteoblasts were transfected with siRNAs and control scrambled RNA using the RNAiFect transfection reagent (Qiagen, Crawley, UK) as per manufacturer's protocol. Briefly, siRNA or scrambled RNA solutions were prepared 15-25 min before the cell transfection. The ratio of siRNA to the RNAiFect reagent was 1 μg siRNA to 3 μl transfection reagent. The siRNA-RNAiFect transfection complexes were incubated for 15 min at room temperature (15-25°C). Osteoblast growth medium was replaced with fresh medium and the siRNA-RNAiFect suspension was added drop-wise onto the cells. The cells with adherent complexes were incubated for 24 h at 37°C, 5% CO_2_, at which point the medium was changed and experimentation was commenced. Transfection efficiency was determined in three preliminary experiments in which a fluorescent control RNA-RNAiFect complex (Qiagen, Crawley, UK) was transfected into the cells instead of the siRNA-RNAiFect complex. The uptake of the fluorescent RNA as determined by fluorescence microscopy was in the range of 75-85%. The ratio of siRNA to the RNAiFect reagent was determined three preliminary experiments with a ratio of 1 μg siRNA:3 μl RNAiFect providing a maximal gene silencing of 75% knockdown as determined by qRT-PCR.

### Quantitative Real Time PCR

Dkk-1 mRNA regulation in primary human osteoblasts treated with dexamethasone was measured by quantitative Real Time PCR using human Dkk-1 QuantiTect assay (Qiagen, Crawley, UK). The QuantiProbe sequence for Dkk-1 was 5'-CACACCAAAGGACAAGA-3'. The forward primer sequence for Dkk-1 was 5'-GGGAATTACTGCAAAAATGGAATA-3', and the reverse primer sequence was 5'-ATGACCGGAGACAAACAGAAC-3'. Total RNA was extracted by TRI-reagent/chlorophorm method, then assayed in duplicate using a Rotorgene 3.0 Real Time PCR instrument (Corbett Research, Cambridge, UK) and the Real Time PCR amplification kit SYBR Green I (Qiagen, Crawley, UK). Using gene specific primer pairs, Dkk1 gene products were measured by Absolute Quantification and were reported as a function of crossing time (Ct), the cycle number at which PCR amplification becomes linear. mRNA expression was normalized to control and GAPDH expression resulting in Mean Fold Change values or ΔΔCt. Following cycling, to ensure specificity, melt curve analysis was carried out to verify the amplification of PCR products starting at 65°C and ramping to 95°C at 0.1°C/sec. One peak in the melt curve indicated no secondary, non-specific products were formed.

### Dkk1 ELISA

Human Dkk1 ELISA kit (R&D Systems Europe Ltd, Abingdon, UK) was obtained to analyse Dkk1 protein expression on cell supernatant. ELISA was performed in accordance with manufacturer's protocols.

### Statistical Analysis

All experimental data presented were obtained from three independent experiments, each in triplicate. Data were expressed as mean ± SE. Statistical differences were calculated using Student's *t*-test or ANOVA for multiple comparisons. *p *values < 0.05 was considered statistically significant.

## Results

### Dexamethasone exposure in vitro reduces alkaline phosphatase activity deposition in primary human osteoblasts

To study the effects of dexamethasone, on markers of osteoblast differentiation, we exposed primary human osteoblasts to 10^-8 ^M dexamethasone over a 72 h time course. The marker of osteoblast differentiation assessed was alkaline phosphatase activity.

**Figure 1:**
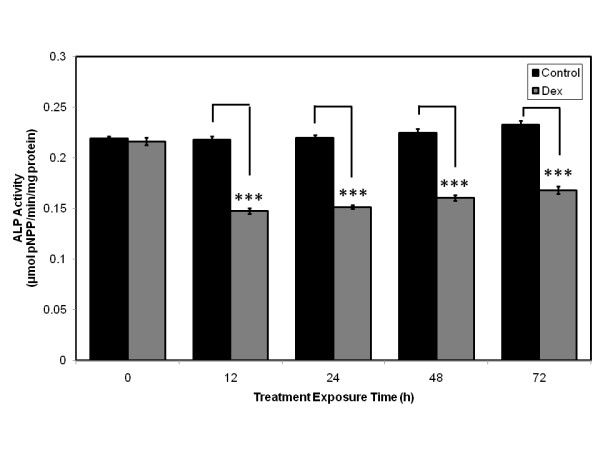
**Primary human osteoblasts exposed to dexamethasone in vitro displaying a reduction in alkaline phosphatase activity over a 72 h time course**. Significant reductions in alkaline phosphatase activity and calcium deposition identified at 12 h, 24 h, 48 h and 72 h of dexamethasone exposure when compared to control primary human osteoblasts. ***p < 0.001 versus control.

Primary human osteoblasts exposed to dexamethasone in vitro displayed a reduction in alkaline phosphatase activity over a 72 h time course (Figure 1). Significant reductions in alkaline phosphatase activity were seen at 12 h (p < 0.001), 24 h (p < 0.001), 48 (p < 0.001), 72 h (p < 0.001; Student's *t*-test) of dexamethasone exposure when compared to control primary human osteoblasts (Figure 1).

### Dexamethasone-induced inhibition of bone turnover in vitro is driven by a Wnt-dependant mechanism

In order to determine the effects of dexamethasone exposure on Wnt/β-catenin signaling in primary human osteoblasts, we firstly examined β-catenin trafficking using immunoflourescence analysis. Primary human osteoblasts were grown to confluency and exposed to 10^-8 ^M dexamethasone over a 48 h time course.

**Figure 2:**
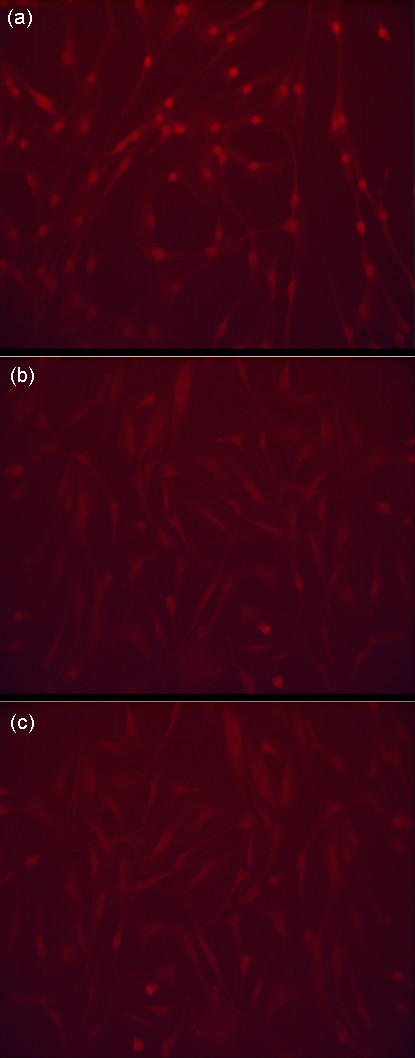
**Immunoflourescence microscopy demonstrating β-catenin expression in primary human osteoblasts exposed to 10^-8 ^M dexamethasone over a 48 h time course**. β-catenin stained with anti-β-catenin antibody demonstrating its perinuclear and intranuclear localization in control cells (a) and a significant reductions in intracellular localization at 24 h (b) and 48 h (c) after treatment.

Control primary human osteoblasts, not exposed to dexamethasone treatment and cultured in osteoblast growth medium alone, demonstrated a strong perinuclear and intranuclear staining of β-catenin, representing activated Wnt/β-catenin signaling (Figure 2a). Primary human osteoblasts exposed to dexamethasone demonstrated a reduction in intracellular staining for β-catenin at 24 h (Figure 2b) and this reduction in β-catenin staining persisted at 48 h (Figure 2c), representing a significant inhibition of Wnt/β-catenin signaling.

**Figure 3:**
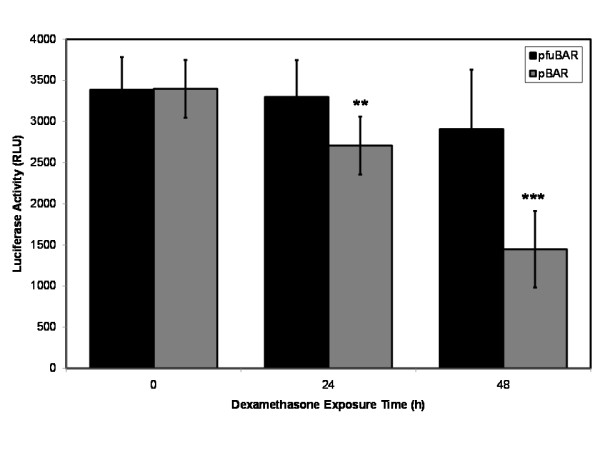
**A reduction in β-catenin expression during dexamethasone treatment results in a reduction in Wnt/β-catenin signaling through TCF/LEF mediated transcription**. Primary human osteoblasts were transiently transfected with pBAR (wild type promoter) and pfuBAR (mutant promoter), and exposed them to 10^-8 ^M dexamethasone over a 48 h time course. Significant reductions in luciferase activity were identified at 24 h and 48 h after treatment in the pBAR cells, whilst luciferase activity in the pfuBAR cells remained unchanged. **p < 0.01, ***p < 0.001 versus pfuBAR.

Since significant changes in intracellular β-catenin trafficking were observed, experiments were performed to elucidate the effects of dexamethasone exposure on Wnt/β-catenin signaling at a transcriptional level. We transiently transfected primary human osteoblasts with the highly sensitive Wnt-luciferase reporter construct pBAR and the control reporter pfuBAR, and exposed them to 10^-8 ^M dexamethasone over a 48 h time course. Significant reductions in luciferase activity were identified at 24 h (p < 0.01) and 48 h (p < 0.01; Student's *t*-test) of dexamethasone treatment in the pBAR reporter cells, and the luciferase activity in the pfuBAR reporter cells remained unchanged during treatment (Figure 3).

**Figure 4:**
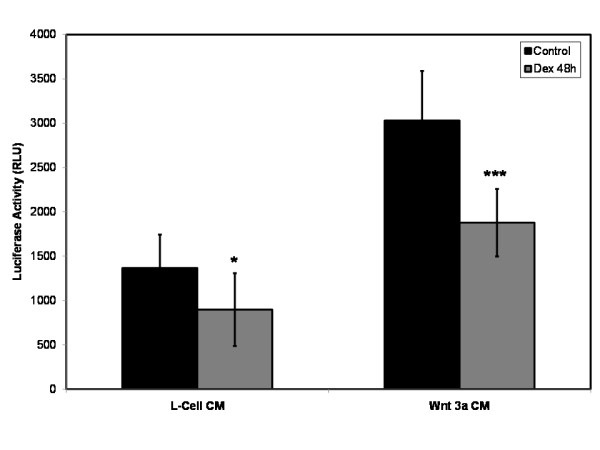
**In order to validate these findings, cells transfected with pBAR were treated with Wnt 3a conditioned media (Wnt agonist) and L-cell conditioned media (control)**. Significant reductions in luciferase activity were identified when 10^-8 ^M dexamethasone was added to both conditioned media over a 48 hr time course. *p < 0.05, ***p < 0.001 versus control.

In order to validate these findings primary human osteoblasts transfected with pBAR were treated with Wnt 3a conditioned media (Wnt agonist) and L-cell conditioned media (control) over a 48 h time course. Significant reductions in luciferase activity were identified when 10^-8 ^M dexamethasone was added to both Wnt 3a conditioned media (p < 0.001) and control L-cell conditioned media (p < 0.05; Student's *t*-test) over the 48 h time course, representing a reduction in Wnt/β-catenin signaling through the inhibition of TCF/LEF mediated transcription (Figure 4).

### Silencing of Dkk1 expression rescues dexamethasone-induced inhibition of osteoblast differentiation, increasing alkaline phosphatase activity and calcium deposition in primary human osteoblasts

**Figure 5:**
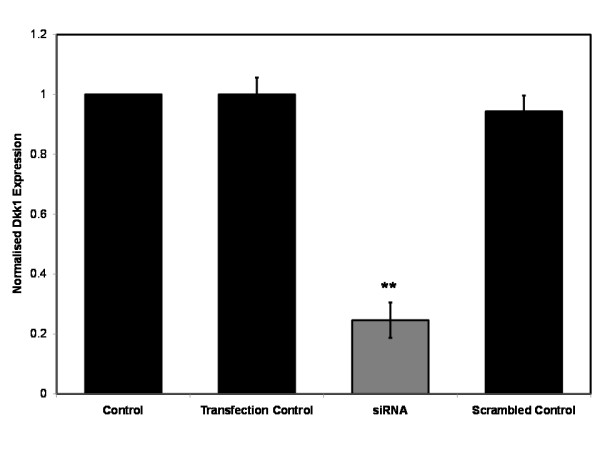
**Primary human osteoblasts transfected with siRNA targeting Dkk1 expression or scrambled control RNA**. Dkk1 gene expression assessed using quantitative RT-PCR to confirm knockdown. **p < 0.01, ***p < 0.001 versus transfection control and scrambled control.

**Figure 6:**
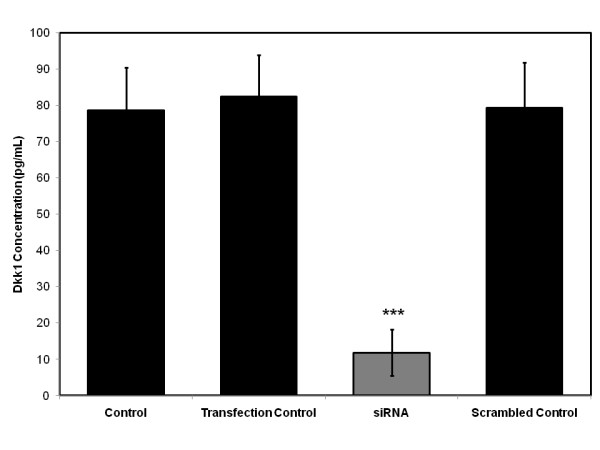
**Primary human osteoblasts transfected with siRNA targeting Dkk1 expression or scrambled control RNA**. Dkk1 protein expression assessed using ELISA to confirm knockdown. **p < 0.01, ***p < 0.001 versus transfection control and scrambled control.

In order to explore the phenotypic effects that aberrations in Wnt/β-catenin signaling have on primary human osteoblasts exposed to dexamethasone, we silenced the expression of Dickkopf-1 (Dkk1), a powerful Wnt antagonist, using siRNA. Primary human osteoblasts were transfected with siRNA targeting Dkk1 expression or scrambled control RNA and Dkk1 gene expression was assessed using quantitative RT-PCR (Figure 5) and ELISA (Figure 6) to confirm knockdown. Quantitative RT-PCR confirmed a 75% reduction in Dkk1 gene expression in the siRNA transfected cells relative to untransfected control cells. This represented a significant reduction when compared to both the scrambled control and transfection control cells (ie. cells treated with RNAiFect alone), which demonstrated no change in Dkk1 gene expression. This reduction in Dkk1 expression was confirmed at protein level when the Dkk1 concentration of cell supernatant was analysed using ELISA. An 85% reduction in Dkk1 concentration was seen in the cell supernatant of siRNA transfected cells relative to untransfected control cells. This also represented a significant reduction when compared to both scrambled control and transfection control cells.

**Figure 7:**
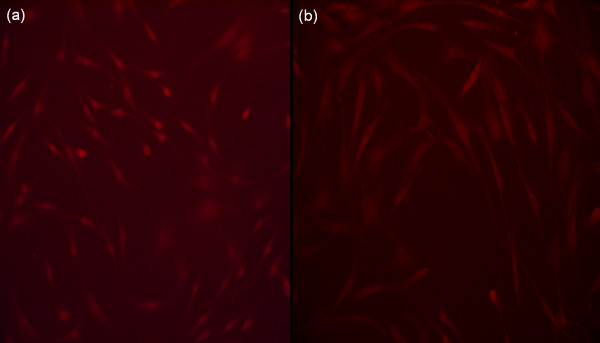
**Immunoflourescence microscopy demonstrating β-catenin expression in primary human osteoblasts transfected with scrambled control RNA (a) or siRNA targeting Dkk1 expression (b)**. siRNA transfected primary human osteoblasts demonstrated a strong perinuclear and intranuclear staining of β-catenin, in comparison to scrambled control primary human osteoblasts. This represents activation of Wnt/β-catenin signaling in response to silencing Dkk1 expression.

In order to confirm that knockdown of Dkk1 was inhibiting Wnt/β-catenin signaling in primary human osteoblasts, we firstly examined β-catenin trafficking using immunoflourescence analysis. Primary human osteoblasts were grown to confluency and transfected with siRNA targeting Dkk1 expression or scrambled control RNA. siRNA transfected primary human osteoblasts (Figure 7a) demonstrated a strong perinuclear and intranuclear staining of β-catenin, in comparison to scrambled control primary human osteoblasts (Figure 7b). This represents activation of Wnt/β-catenin signaling in response to silencing Dkk1 expression.

**Figure 8:**
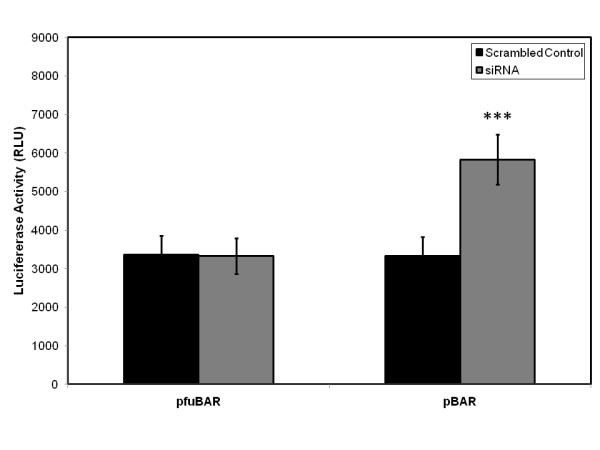
**This increase in β-catenin expression by silencing Dkk1 expression results in an increase in Wnt/β-catenin signaling through TCF/LEF mediated transcription**. Primary human osteoblasts were transiently transfected with pBAR (wild type promoter) and pfuBAR (mutant promoter), and subsequently treated with siRNA targeting Dkk1 expression or scrambled control RNA. A significant increase in luciferase activity was observed in the siRNA transfected cells when compared to scrambled control cells in the pBAR reporter cells, whilst luciferase activity in the pfuBAR cells remained unchanged (c). ***p < 0.001 versus scrambled control.

These changes in intracellular β-catenin trafficking were accompanied by changes in Wnt/β-catenin signaling at a transcriptional level. Primary human osteoblasts transfected with Wnt-luciferase reporter construct pBAR and the control reporter pfuBAR, were subsequently treated with siRNA targeting Dkk1 expression or scrambled control RNA. A significant increase in luciferase activity was observed in the siRNA transfected cells when compared to scrambled control cells (p < 0.001; Student's *t*-test) in the pBAR reporter cells, whilst the luciferase activity in the pfuBAR reporter cells remained unchanged during treatment (Figure 8).

**Figure 9:**
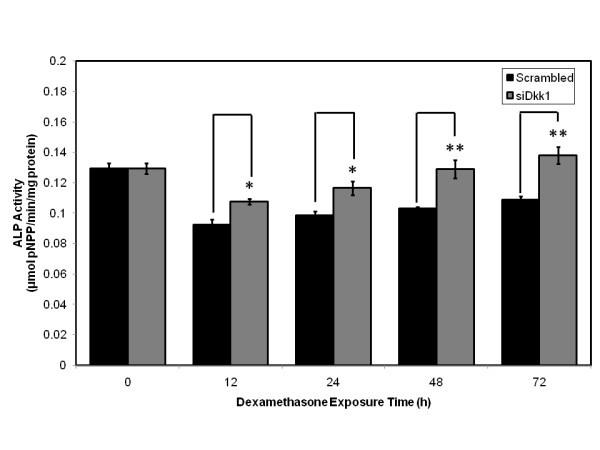
**Primary human osteoblasts transfected with siRNA and scrambled control exposed to dexamethasone in vitro compared with respect to alkaline phosphatase activity over a 72 h time course**. Significant increases in alkaline phosphatase activity relative to scrambled control identified at 12 h, 24 h, 48 h and 72 h of dexamethasone exposure. *p < 0.05, **p < 0.01 versus scrambled control.

Primary human osteoblasts transfected with siRNA targeting Dkk1 expression or scrambled control RNA were subsequently exposed to 10^-8 ^M dexamethasone over a 72 h time course. Bone turnover was assessed by analyzing alkaline phosphatase activity. Control primary human osteoblasts exposed to dexamethasone over the 72 h time course displayed reductions in alkaline phosphatase activity at 12 h, 24 h, 48 h and 72 h. However, when primary human osteoblasts were transfected with siRNA attenuating Dkk1 expression, there was an increase in alkaline phosphatase activity at each individual time point when compared to scrambled control. Significant increases relative to scrambled control were identified at 12 h (p < 0.05), 24 h (p < 0.05), 48 h (p < 0.01) and 72 h (p < 0.01; Student's *t*-test) of dexamethasone exposure (Figure 9).

## Discussion

Osteoporosis is a skeletal disorder characterized by low bone mass and progressive micro-architectural deterioration of bone tissue with a consequent increase in bone fragility and propensity to fracture. Reduced bone mass is a result of an imbalance between the tightly regulated processes of bone formation and bone resorption, controlled by osteoblasts and osteoclasts respectively. There have been many cell signaling cascades linked to bone mass regulation. One pathway seen as critical for bone mass accrual, bone remodeling and even fracture repair is the Wnt/β-catenin pathway.

The Wnt/β-catenin pathway plays an important role in the development and maintenance of many tissues and organs, with disturbances in Wnt signaling implicated in multiple disease processes [5,12]. Recent evidence has implicated the Wnt β-catenin pathway as a major signaling cascades in bone biology [13-17]. Human genetic studies have revealed a critical role of the Wnt/β-catenin pathway in bone mass acquisition and maintenance [18]. Loss of function of the Wnt co-receptor, LRP5, have been shown to lead to disorders of decreased bone mass, such as Osteoporosis Pseudoglioma Syndrome (OPS) [7]. Whereas, gain of function mutations of LRP5 have been shown to lead to disorders of increased bone mass such as sclerostosis [19], Van Buchem's disease [20], osteopetrosis [9] and endosteal hyperostosis [10].

The Dkk family of extracellular proteins consists of four members (Dkk1, Dkk2, Dkk3 and Dkk4). Dkk1 and Dkk2 are the best characterized members of this family. Dkk1 has been shown to inhibit canonical Wnt/β-catenin signaling pathway in several different organisms and cell types [21-25]. The involvement of Dkk2 in regulating bone development was recently demonstrated when it was shown that knockout mice lacking Dkk2 developed osteopenia [26]. Initially increased Dkk1 expression was found to be associated with the lytic bone lesions in patients with multiple myeloma, suggesting that Dkk1 might inhibit osteoblast differentiation or function [27]. This has been subsequently been reinforced by data showing Dkk1 to be a powerful negative regulator of osteoblast function *in vitro *and *in vivo *and that a decrease in its expression is sufficient to induce a strong anabolic response in the skeleton [28-30].

Our data emphasizes the key role of Wnt/β-catenin signaling in regulating bone development and remodeling, further strengthening its crucial role in glucocorticoid-induced osteoporosis [31-33]. Primary human osteoblasts exposed to dexamethasone *in vitro *display a reduction in alkaline phosphatase activity over a 72 h time course when compared to control osteoblasts. These phenotypic changes are driven by a Wnt/β-catenin-dependent mechanism as clearly demonstrated by both alterations in β-catenin trafficking and changes in TCF/LEF-mediated transcription.

Dkk1 is a powerful antagonist of canonical Wnt/β-catenin signaling. Silencing Dkk1 expression rescues dexamethasone-induced suppression of primary human osteoblast function. Increased alkaline phosphatase activity was displayed by primary human osteoblasts treated with siRNA targeting Dkk1 expression when compared to scrambled control cells over a 72 h time course of dexamethasone exposure. The pharmacological targeting of the Wnt/β-catenin signaling pathway offers an exciting opportunity for the development of novel anabolic bone agents to treat osteoporosis and disorders of bone mass.

## Conclusion

Wnt/β-catenin signaling plays a key role in regulating glucocorticoid-induced osteoporosis *in vitro*. Silencing Dkk1 expression rescues dexamethasone-induced suppression of primary human osteoblast differentiation. Targeting of the Wnt/β-catenin signaling pathway offers an exciting opportunity to develop novel anabolic bone agents to treat osteoporosis and disorders of bone mass.

## Competing interests

The authors declare that they have no competing interests.

## Authors' contributions

JSB performed the majority of the research work. JMQ, BMD and DWM participated in study design, data interpretation and drafted the manuscript. PPD and JMO'B provided direction and oversight regarding all aspects of study design and interpretation of results. All authors have read and approved the final manuscript.

## Pre-publication history

The pre-publication history for this paper can be accessed here:

http://www.biomedcentral.com/1471-2474/11/210/prepub
